# Correlation between Serum Tryptase, Mast Cells Positive to Tryptase and Microvascular Density in Colo-Rectal Cancer Patients: Possible Biological-Clinical Significance

**DOI:** 10.1371/journal.pone.0099512

**Published:** 2014-06-10

**Authors:** Michele Ammendola, Rosario Sacco, Giuseppe Sammarco, Giuseppe Donato, Severino Montemurro, Eustachio Ruggieri, Rosa Patruno, Ilaria Marech, Marica Cariello, Angelo Vacca, Cosmo Damiano Gadaleta, Girolamo Ranieri

**Affiliations:** 1 Department of Medical and Surgery Science, Clinical Surgery Unit, University of Catanzaro “Magna Graecia” Medical School, Catanzaro, Italy; 2 Surgery Unit, National Cancer Research Centre Istituto Tumori “Giovanni Paolo II”, Bari, Italy; 3 Department of Medical and Surgery Science, Pathology Unit, University of Catanzaro “Magna Graecia” Medical School, Catanzaro, Italy; 4 Department of Health, ASL BAT, Bari, Italy; 5 Interventional Radiology Unit with Integrated Section of Translational Medical Oncology, National Cancer Research Centre Istituto Tumori “Giovanni Paolo II”, Bari, Italy; 6 Department of Biomedical Science and Human Oncology Clinica Medica “G. Baccelli”, University of Bari Medical School, Bari, Italy; University Hospital Carl Gustav Carus Dresden, Germany

## Abstract

**Background:**

Tryptase is a serin protease stored and released from mast cells (MCs) that plays a role in tumour angiogenesis. In this study we aimed to evaluate serum tryptase levels in colo-rectal cancer (CRC) patients before (STLBS) and after (STLAS) radical surgical resection. We also evaluated mast cell density positive to tryptase (MCDPT) and microvascular density (MVD) in primary tumour tissue.

**Methods:**

A series of 61 patients with stage B and C CRC (according to the Astler and Coller staging system) were selected. Serum blood samples were collected from patients one day before and one day after surgery. Tryptase levels were measured using the UniCAP Tryptase Fluoroenzymeimmunoassay (Pharmacia, Uppsala, Sweden). Tumour sections were immunostained with a primary anti-tryptase antibody (clone AA1; Dako, Glostrup, Denmark) and an anti CD-34 antibody (QB-END 10; Bio-Optica Milan, Italy) by means of immunohistochemistry and then evaluated by image analysis methods.

**Results:**

The mean ± s.d. STLBS and STLAS was 5.63±2.61 µg/L, and 3.39±1.47 µg/L respectively and a significant difference between mean levels was found: p = 0.000 by t-test. The mean ± s.d. of MCDPT and MVD was 8.13±3.28 and 29.16±7.39 respectively. A strong correlation between STLBS and MVD (r = 0.83, p = 0.000); STLBS and MCDPT (r = 0.60, p = 0.003); and MCDPT and MVD (r = 0.73; p = 0.001) was found.

**Conclusion:**

Results demonstrated higher STLBS in CRC patients, indicating an involvement of MC tryptase in CRC angiogenesis. Data also indicated lower STLAS, suggesting the release of tryptase from tumour-infiltrating MCs. Serum tryptase levels may therefore play a role as a novel bio-marker predictive of response to radical surgery. In this context tryptase inhibitors such as Gabexate and Nafamostat Mesilate might be evaluated in adjuvant clinical trials as a new anti-angiogenic approach.

## Introduction

Angiogenesis is a complex process involved in the growth, invasion, and metastasis of several human and animal tumours [1]–[3]. Mast Cells (MCs) intervene in this process releasing classical pro-angiogenic factors, such as vascular endothelial growth factor (VEGF), thymidine phosphorylase (TP), fibroblast growth factor-2 (FGF-2), and interleukin-6 (IL-6), as well as non-classical pro-angiogenic factors, such as tryptase, stored in their secretory granules [4]–[8]. Data from experimental tumour models suggest that MCs accumulate near to tumour cells before the onset of angiogenesis and that they are required for the macroscopic expansion and metastatic spread of primary tumour cells. Evidence for a specific role for MCs has been reported in animal and human cancers, such as MC tumours, head and neck, gastric, colo-rectal, lung and cutaneous malignancies, where MC density is highly correlated with the extent of tumor angiogenesis [9]–[19].

Tryptase is a neutral serine protease with a molecular weight of 134 kDa and a tetrameric structure consisting of non-covalently linked subunits. Four different forms of tryptase have been identified in human MCs thus far: α-, β-, γ- and δ-tryptase [20]. Of these, α- and β-tryptase are the two best circulating isoforms described. α-tryptase is constantly released from MCs in the bloodstream. β-tryptase is selectively concentrated in the secretory granules of MCs and is released only after degranulation. The presence of a high concentration of β-tryptase in the bloodstream is therefore a clear expression of MC activation [21]–[23]. Experimental studies have shown the release of tryptase from MCs to be a potent angiogenic stimulus which is able to induce neo-vascolarization both in *vitro and in vivo* and is therefore involved in cancer development.

In this pilot study, we analyzed serum tryptase levels (STLs) in CRC patients before (STLBS) and after (STLAS) radical surgical resection; MC density positive to tryptase (MCDPT); and microvascular density (MVD) in primary tumour tissue in order to correlate each to the others and to evaluate STLs as a possible bio-marker predictive of response to radical surgery. In this context, the inhibition of tryptase by means of gabexate and nafamostat may be evaluated in adjuvant clinical trials as a new anti-cancer therapy [24]–[26].

## Materials and Methods

### Study Populations

A series of 61 CRC patients observed at the Clinical Surgery Unit of the “Magna Graecia” University of Catanzaro were selected. Helical computed tomography of the thorax, abdomen and pelvis revealed that no patients had distant metastases. Various surgical approaches were used: left and right open hemicolectomy for colon cancer; open anterior resection, total meso-rectal excision and open abdomino-perineal resection for rectal cancer [27]–[30]. Patients with stage B and C CRC according to the Astler and Coller staging system were enrolled. In the global series there were 61 adenocarcinomas; the histopathological grading was performed according to the AJCC 7^th^ Edition [31]–[32]. The clinico-pathological features of the patients are summarized in Table 1. Full ethical approval and signed consent from individual patients were obtained to conduct the study. The full name of ethics institutional committee review board that approved our study is: University Hospital Ethics Committee “Mater Domini”, Germaneto, Catanzaro, Italy.

**Table 1:** Clinico-pathological features of patients.

Variable	Number of cases
***Overall series***	**61**
***Gender***	
** Male**	**37**
** Female**	**24**
***Tumour site***	
** Colon**	**43**
** Rectal**	**18**
***Astler-Coller staging system***	
** B**	**19**
** C**	**42**
***Histologic type***	
** Adenocarcinomas**	**61**
***Histologic grade***	
** G1-2**	**38**
** G3**	**23**

### Sample preparation

Blood samples were collected between 7:00 and 9:00 a.m. after overnight fasting, one day before and one day after surgical resection of the tumour. The samples were immediately dispensed into test tubes with serum separator tubes without additives (Becton Dickinson Vacutainer Systems Hemogard, Plymouth, UK) and left for at least 30 min at room temperature to allow for a complete clotting process. The samples were subsequently centrifuged at 1,500×g for 15 min at room temperature and the supernatant was recovered. Patient sera obtained were collected, aliquoted and frozen at −80°C until the analysis phase.

Prior to starting the analytical phase, the samples were thawed to room temperature and mixed thoroughly by vortexing at low speed in order to eliminate any residues of fibrin or other particulate matter potentially affecting reproducible results. Lipemic or hemolyzed samples potentially interfering with the assay were excluded from the analytical evaluation.

Serum tryptase levels were measured by fluoro-enzyme-immunoassay (FEIA) for each sample, using Uni-CAP100 (Pharmacia Diagnostics AB, Uppsala, Sweden).

### Immunohistochemistry

For the evaluation of MCPT and MVD a three-layer biotin-avidin-peroxidase system was utilized [33]. Briefly, 4 µm-thick serial sections of formalin-fixed and paraffin-embedded tumour sample and adjacent normal mucosa were cut. Sections were then microwaved at 500 W for 10 min, after which endogenous peroxidise activity was blocked with 3% hydrogen peroxide solution. Tumour sections were incubated with an anti-tryptase (clone AA1; Dako, Glostrup, Denmark) diluted 1∶100 for 1 h at room temperature and an anti-CD34 antibody (QB-END 10; Bio-Optica Milan, Italy) diluted 1∶50 for 1 h at room temperature as a pan-endothelial marker respectively. Adjacent normal mucosa sections were incubated with an anti-tryptase (clone AA1; Dako, Glostrup, Denmark) and then processed in the above manner. Tumour sections were also incubated with an anti-VEGF (Clone VG-1) (Dako Glostrup, Denmark), diluted to 1∶200 for 1 h at room temperature. The bound antibody was visualized using a biotinylated secondary antibody, avidin-biotin peroxidise complex and fast red. Nuclear counterstaining was performed with Gill's haematoxylin no. 2 (Polysciences, Warrington, PA, USA). The primary antibody was omitted in negative controls.

### Morphometrical assay

**Figure 1:**
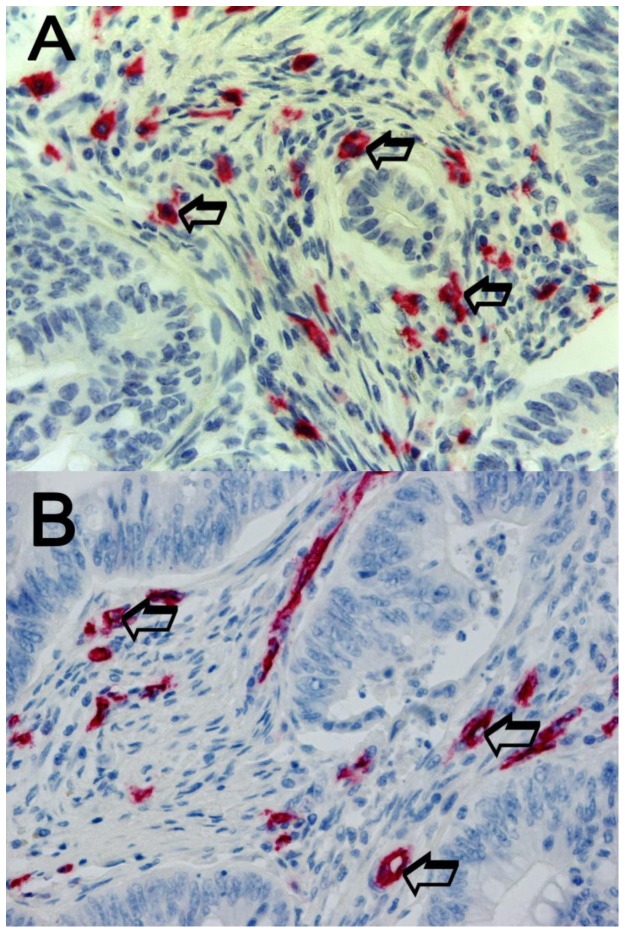
Colon cancer sections immunostained for the evaluation of MCDPT and MVD at x400 magnification, high MCDPT and MVD respectively. In **A** a primary anti-tryptase antibody has been employed. Arrows indicate red mast cells positive to tryptase. In **B** a primary anti CD-34 antibody has been employed. Arrows indicate red positive microvessels.

**Figure 2:**
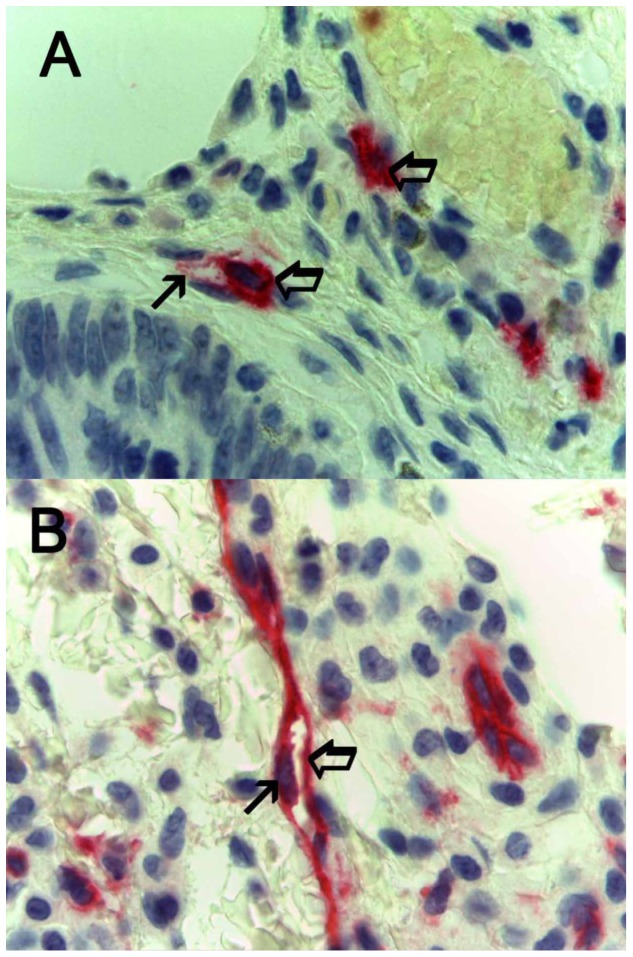
Colon cancer sections immunostained for the detail evaluation of MCDPT and MVD at x1000 magnification in oil respectively. In **A** a primary anti-tryptase antibody has been employed. The big arrows indicate single red mast cells positive to tryptase. The small arrow indicates the degranulation front of the mast cell forming a microvessel. In **B** a primary anti CD-34 antibody has been employed. The big arrow indicates a red positive microvessel. The small arrow indicates the nucleus of the endothelial cell. Note the lumen of the microvessel.

**Figure 3:**
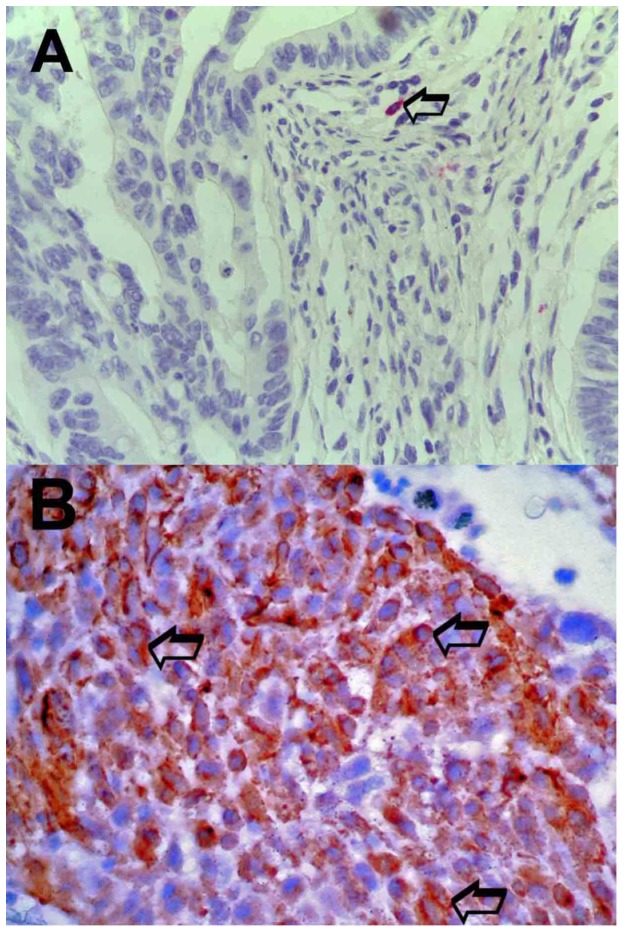
Adjacent normal mucosa section and colon cancer section immunostained for the evaluation of MCDPT and VEGF expression at x400 magnification respectively. In **A** a primary anti-tryptase antibody has been employed. Arrow indicates a single and unique red mast cells positive to tryptase in the observed field. In **B** a primary anti-VEGF antibody has been employed. Many red immunostained cancer cells. Big arrows indicate single red cytoplasmic cancer cells positive to VEGF.

An image analysis system (Quantimet500 Leica, Wetzlar, Germany) was utilized [34]. In tumour sections five most immunostained areas (hot spots) were selected at low magnification and individual MCDPT (Fig. 1A) and MVD (Fig. 1B) were counted at x400 magnification (0.19 mm^2^ area). The details were evaluated at x1000 magnification in oil (Figs. 2A and 2B respectively). In adjacent normal mucosa sections five most immunostained areas (hot spots) were selected at low magnification and individual MCDPT (Fig. 3A) were counted at x400 magnification (0.19 mm^2^ area). In tumour tissue VEGF expression was evaluated (Fig 3B).

### Statistical analysis

MCDPT and MVD mean values ±1 standard deviations (SD) were evaluated by two independent observers (G.R. and G.D.) for each tumour sample and in all series of sections. MCDPT mean values ±1 SD was also evaluated for each adjacent normal mucosa. Correlations between STLs, MCDPT, and MVD were calculated using Pearson's (r) analysis. The correlations between the above indexes and the clinico-pathological features listed in Table 1 were analyzed by the Chi-square test. All statistical analyses were performed with the SPSS statistical software package (SPSS, Inc., Chicago, IL).

## Results

**Figure 4:**
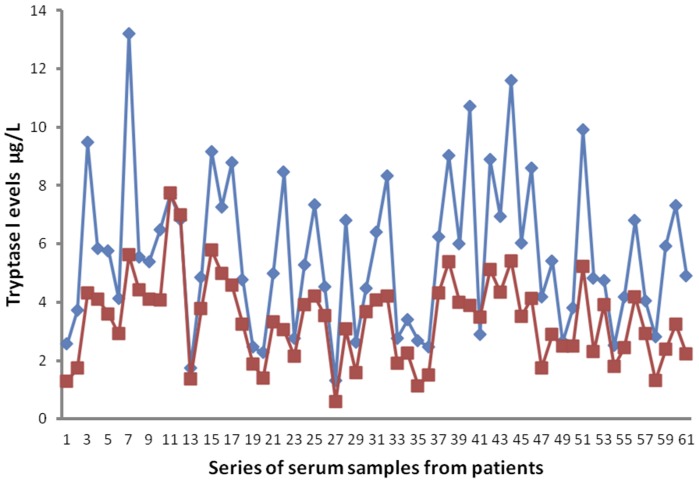
Differences between STLBS (red line) and STLAS (blu line).

**Figure 5:**
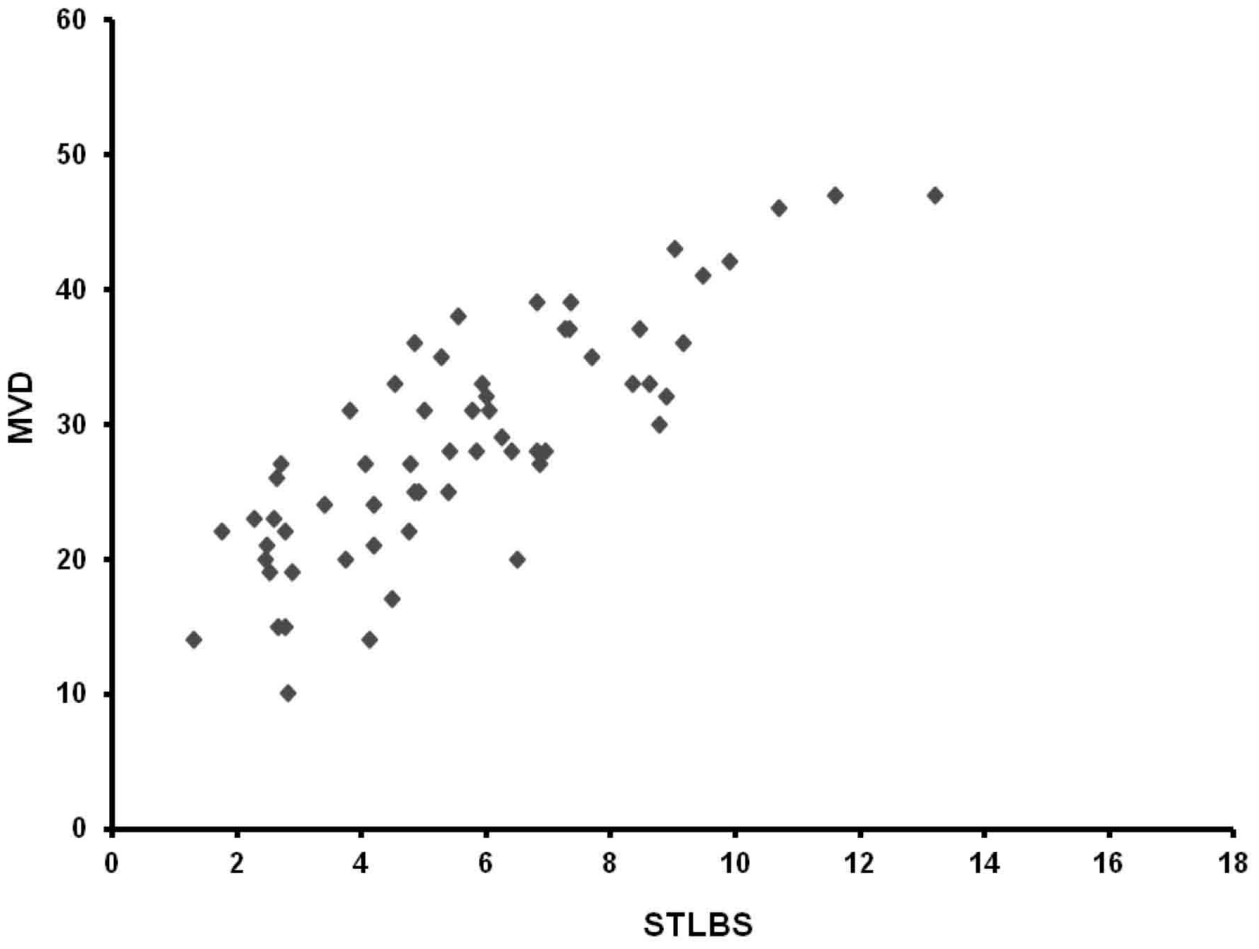
Correlation analysis between MVD and STLBS.

**Figure 6:**
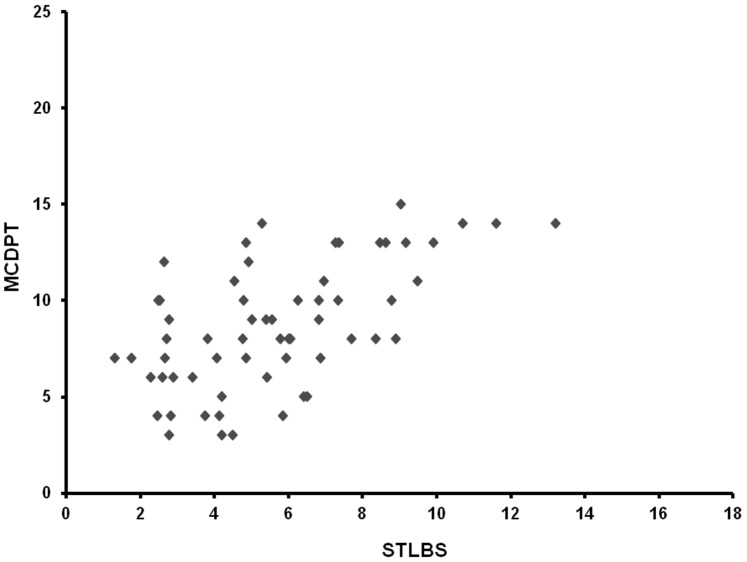
Correlation analysis between MCDPT and STLBS.

**Figure 7:**
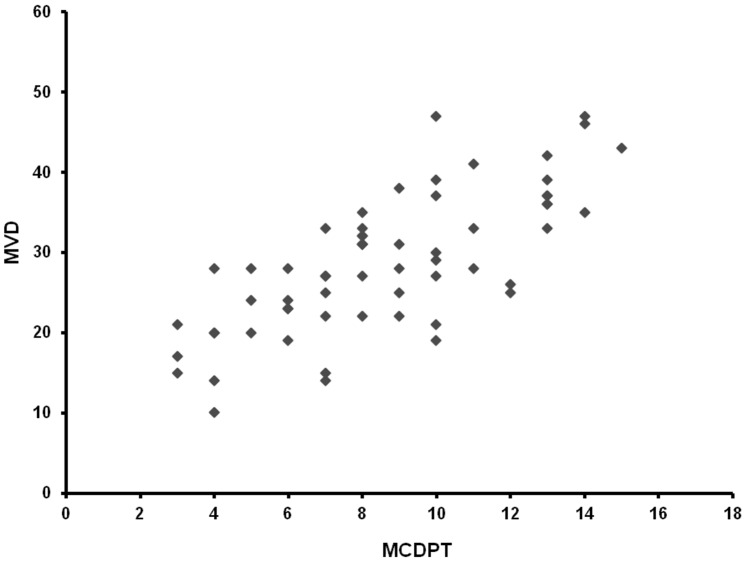
Correlation analysis between MVD and MCDPT.

The mean ± s.d. STLBS and STLAS were 5.63±2.61 µg/L and 3.39±1.47 µg/L respectively, and a significant difference between mean levels was found: p = 0.000 by t-test (Table 2, Fig. 4). In tumour tissue the mean ± s.d. of MCDPT and MVD was 8.13±3.28 and 29.16±7.39 respectively (Table 2). A significant correlation between STLBS and MVD (r = 0.83, p = 0.000, Fig. 5), STLBS and MCDPT (r = 0.60, p = 0.003, Fig. 6), and MCDPT and MVD (r = 0.73; p = 0.001, Fig. 7) was found. In adjacent normal mucosa the mean ± s.d. of MCDPT was 2,63±1,17 (Fig. 3A). A significant difference between tumour tissue MCDPT mean and adjacent normal mucosa MCDPT mean was found (p = 0.000). VEGF expression showed a strong cytoplasmic immunostaining in cancer tissue (Fig. 3B), however based to previously published data demonstrating a strong expression of VEGF in CRC tissue and blood fraction from patients as compared to adjacent normal mucosa and healthy controls respectively data were not shown [35]–[38].

**Table 2:** STLBS, STLAS, MCDPT and MVD means ±1 standard deviations in a series of 61 colo-rectal cancer patients.

*STLBS µg/L*	*STLAS µg/L*	*MCDPT in Tumour Tissue*	*MVD in Tumour Tissue*	*MCDPT in Normal Mucosa*
		X400 magnification (0.19 mm^2^ area)	X400 Magnification (0.19 mm^2^ area)	X400 magnification (0.19 mm^2^ area)
5,63±2,61^a^	3,39±1,47^a^	8,13±3,28^a^	29,16±7,39^a^	2,63±1,17^a^

a Mean ±1 standard deviation.

No other significant correlations between the above indexes and the main clinico-pathological features listed in table 1 was found.

## Discussion

In this pilot study we have shown for the first time that STLBS strongly correlates with MCDPT and MVD in primary tumour tissue. Our data also demonstrates that STLs decrease following surgical resection in CRC patients with a significantly lower value of STLAS. Due to the release of tryptase from MC activation, we suggest that MCDPT in primary CRC tumour tissue represents the main source of serum tryptase. In our hypothesis, if primary tumour tissue is completely removed STLs should decrease in a day due to their approximately 4-h long life-cycle. For these reasons, we detected STLs 24 h before surgery to evaluate their possible role as a circulating biomarker suggesting the presence of tumour tissue, and again 24 h after treatment to confirm the concentration decrease and, as a consequence, its possible implication of the absence of tumour tissue. We elaborate the background of our hypothesis based on previously published pilot data which suggested an increase of MCDPT in primary tumour tissue, and here our results also confirmed the reported data (Fig 1A, 3A). In these studies STLBS and MCDPT were correlated with MVD, suggesting their role in CRC angiogenesis. Our data agree with a previously published study that demonstrated an involvement of MCs in different malignancies such as hematological malignancies, melanoma, breast cancer, gastrointestinal and colo-rectal cancers, and also in animal tumour models [38]–[43]. However the above studies did not focus on the changes in STLs before and after surgery, and no correlation between STLs, MCDPT and MVD was evaluated. Interestingly, tryptase released from MCs is involved in tumour angiogenesis by several mechanisms: firstly, tryptase stimulates the formation of vascular tubes in *in vitro* and *in vivo* experimental models; secondly, tryptase is an agonist of the proteinase-activated receptor-2 in vascular endothelial cells that, in turn, induces angiogenesis [44]; thirdly, tryptase may stimulate the release of latent angiogenic factors bound to the extracellular matrix [45]–[47]. Considered together, the above data suggest that tryptase may be a potential surrogate bio-marker of tumour angiogenesis which is able to predict response to surgical treatment.

Therefore, with the primary source of tryptase production no longer existing, after 24 h a significant reduction in STLs should be expected. If elevated STLs persist after surgery, this would suggest that residual tumour tissue remains after surgical resection or, alternatively, that unknown metastases are present. In this context, several tryptase inhibitors such as Gabexate or Nafamostat mesilate may be evaluated in future clinical trials as a new anti-tumour and anti-angiogenic approach.
